# A Cube Version of the Square–Diamond Illusion

**DOI:** 10.1177/2041669520986574

**Published:** 2021-01-20

**Authors:** Hiroyuki Ito, Daichi Nose

**Affiliations:** Faculty of Design, 12923 Kyushu University, Minami-ku, Fukuoka, Japan; Research Center for Applied Perceptual Science, Kyushu University, Minami-ku, Fukuoka, Japan; Research and Development Center for Five-Sense Devices, Kyushu University, Nishi-ku, Fukuoka, Japan; Department of Visual Communication Design, 12923 Kyushu University, Minami-ku, Fukuoka, Japan

**Keywords:** angle illusion, square–diamond illusion, Shepard tabletop illusion, oblique effect

## Abstract

The square–diamond illusion is often referred to as a type of size illusion. However, the 45-degree tilting of a square remarkably affects perceived corner angles when a cube version of the figure is used. This illusion is measured and discussed in relation to anisotropy in shape interpretation.

A square is perceived as a *diamond* when tilted at 45 degrees as shown in Figures 1A and B (Schumann, 1900). The *square* and the *diamond* are geometrically congruent, but not recognized as such (Mach, 1897). This square–diamond illusion is essentially a shape illusion but has been referred to as a size illusion (i.e., the perceived *diamond* seems bigger than the *square*). In fact, the *diamond* does not look as a shape like a diamond printed on cards (see Figure 1C), where the top and bottom corners are acute while the left and right corners are obtuse. This article shows that a corner–angle illusion becomes obvious when a cube version of the square–diamond illusion figure is used. In Figure 2, the two figures are precisely the same except that the right figure is rotated at 45 degrees in a counter-clockwise direction. Comparing with Figure 1B, the surface *b* is perceived more like a diamond printed on cards while the surface *a* is perceived as a square. We measured the illusion and discuss the results together with related phenomena.

**Figure 1.**
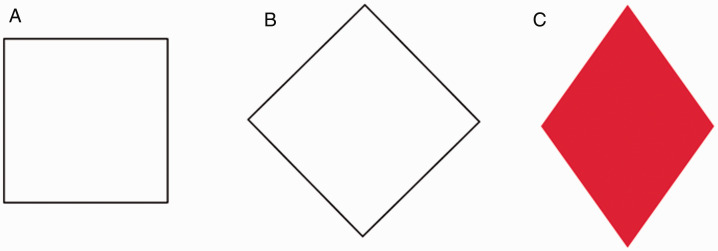
Square–diamond illusion (A and B) and a diamond on cards (C).

**Figure 2.**
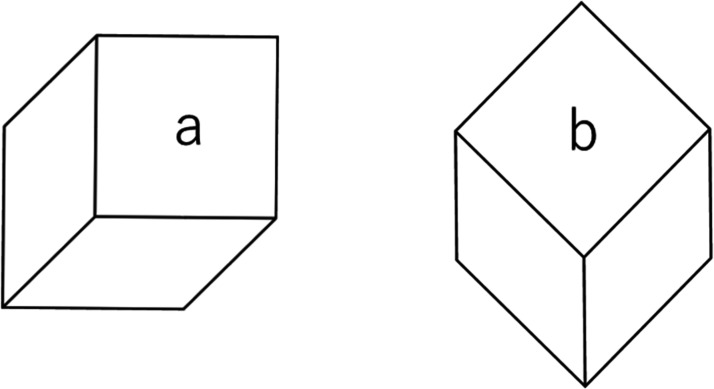
Cube version of the square–diamond illusion.

Stimuli were created on a computer (Dell, Inspiron 580s) and displayed on an organic light-emitting-diode display (Sony, PVM-2541; see Ito et al., 2013). The display resolution was a matrix of 1920 × 1080 pixels. The stimuli were drawn as black anti-aliased lines (0.0 cd/m^2^) with a 3-pixel width on a white background (39.4 cd/m^2^). Figure 3 shows the stimuli tested. Despite having different orientations, all the cube figures were physically the same. One side of the square subtended 3.7 degrees in visual angle from a viewing distance of 60 cm. Perceived corner angles were individually measured using a matching method (Figure 4). A total of 160 trials (10 figures × 4 corners × 4 repetitions) were performed in random order. A chinrest was used to avoid head tilt. Eye movements were not restricted. Ten observers participated in the experiment.

**Figure 3.**
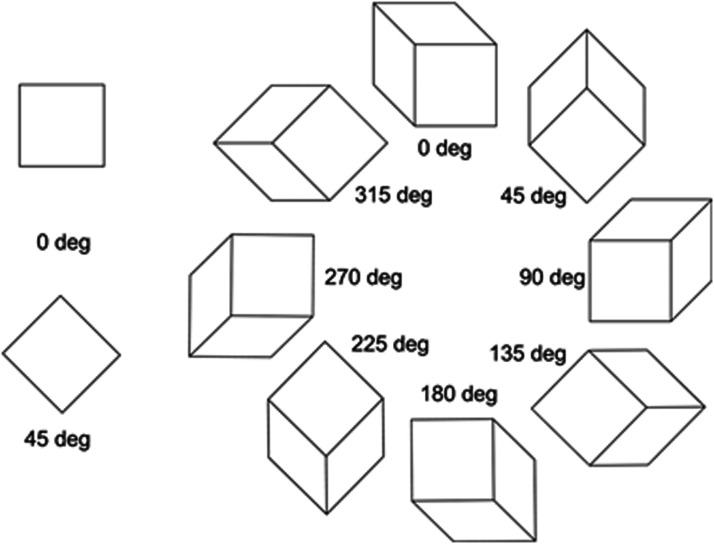
Stimulus figures tested.

**Figure 4.**
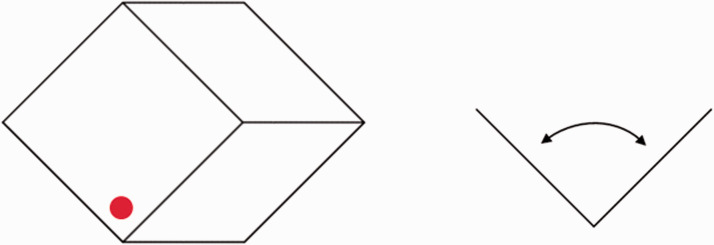
Measurement of a perceived corner angle. A stimulus figure was presented for 2.0 s with a red marker presented for the first 1.0 s to indicate a targeted corner (left panel). After a blank period of 1.0 s, matching line segments appeared at a 10.8-degree rightward position. Observers used keys to control the angle of the line segments in 1-degree steps (right panel). Observers could view the stimulus repeatedly. The orientation of the matching line segments was matched to the targeted-corner orientation.

For the normal square–diamond illusion figures, there was evidence in the *diamond* for a systematic sharpening of all angles (see Figure 5). The perceived angles were significantly smaller than 90 degrees for corners L’ (*p* = .0083), T’ (*p* = .0022), R’ (*p* = .0044), and B’ (*p* = .0911). Aside from the perceived size change as reported in Ninio (2015), a tilt of 45 degrees appears to result in some perceptual sharpening of corner angles. In contrast, there was a greater effect for cube versions at tilts of 45, 135, 225, and 315 degrees. As an example, at a tilt of 315 degrees, the matched angle was 84.8 degrees on average for corner *a* and 93.1 degrees on average for corner *b*; thus, the perceived shape may be that of a horizontally elongated diamond rather than that of a simply tilted square.

We defined a *diamond index*—that is, (*b*’+*d*’)/2−(*a*’+*c*’)/2—as the difference between the average of perceived angles for *a* and *c* and the average of perceived angles for *b* and *d* (see Figures 5 and 6). When all corner angles are perceived the same, the index is zero. When the perceived shape is elongated along the *a*–*c* or *b–d* diagonal axis, the index is positive or negative, respectively. We analyzed the index adopting a one-way analysis of variance. The Greenhouse–Geisser epsilon was used to correct for the degree of freedom as the violation of sphericity was significant (*p* < .01). The effect of orientation was significant—*F*(2.55, 22.94) = 20.6057, *p* < .0001, ηp^2^ = 0.6960). Multiple comparisons (alpha = .05) revealed that the index for a tilt of 135 or 315 degrees was greater than that for a tilt of 0, 90, 180, or 270 degrees, that the index for a tilt of 225 degrees was greater than that for a tilt of 0, 90, or 270 degrees, and that the index for a tilt of 45 degrees was greater than that for a tilt of 0 or 90 degrees. No significant difference was found for pairs among tilts of 0, 90, 180, and 270 degrees or among tilts of 45, 135, 225, and 315 degrees. When the cube figure was rotated through 45, 135, 225, or 315 degrees, corners *a* and *c* were largely underestimated while corners *b* and *d* were overestimated; that is, the square was perceptually elongated along the *a*–*c* diagonal axis.

**Figure 5.**
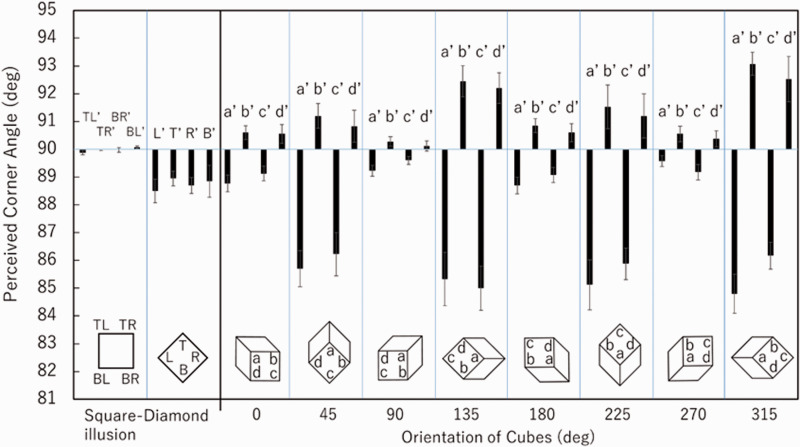
Results of the experiment. *a*’, *b*’, *c*’ or *d*’ denotes the perceived angle for *a*, *b*, *c* or *d*, respectively. Error bars indicate standard errors.

**Figure 6.**
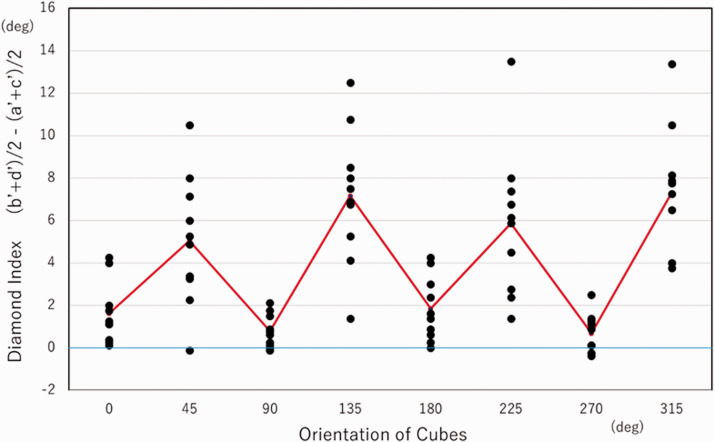
Diamond indexes. Symbols indicate data for different observers. The red line indicates averaged values.

Perceived vertical elongation in the recovery of the depth dimension may cause the Shepard tabletop illusion (Shepard, 1981; Tyler, 2011) or an angle illusion for a straight road (Osa et al., 2011). However, all the present cube figures could be interpreted to have a surface slanted in depth. Anisotropy in the interpretation of the figure is thus the essence of this illusion. The squares with tilts of 0, 90, 180, and 270 degrees comprise horizontal and vertical sides, which are considered to be precisely processed and to be robust as a spatial frame of reference, compared with the weak processing for oblique sides (Appelle, 1972; Goldmeier, 1972). This could disturb the perceptual slant and elongation of the upright square along an oblique *a*–*c* axis. The squares tilted through 45, 135, 225, and 315 degrees have oblique sides. Thus, there could be room to perceptually elongate the tilted squares in the recovery of the depth dimension. The perceptual elongation occurred equally in horizontal and vertical dimensions according to the *a*–*c* axis orientation. In the case of the present effect, the same figures in different orientations may have different depth interpretations, resulting in a corner-angle illusion. While squares with horizontal and vertical sides are robust, squares with oblique sides are easily deformed in perception.
